# Perceptual learning in patients with macular degeneration

**DOI:** 10.3389/fpsyg.2014.01189

**Published:** 2014-10-17

**Authors:** Tina Plank, Katharina Rosengarth, Carolin Schmalhofer, Markus Goldhacker, Sabine Brandl-Rühle, Mark W. Greenlee

**Affiliations:** ^1^Institute for Experimental Psychology, University of RegensburgRegensburg, Germany; ^2^Department of Ophthalmology, University Medical Center RegensburgRegensburg, Germany

**Keywords:** perceptual learning, fMRI BOLD, cortical plasticity, visual cortex, macular degeneration

## Abstract

Patients with age-related macular degeneration (AMD) or hereditary macular dystrophies (JMD) rely on an efficient use of their peripheral visual field. We trained eight AMD and five JMD patients to perform a texture-discrimination task (TDT) at their preferred retinal locus (PRL) used for fixation. Six training sessions of approximately one hour duration were conducted over a period of approximately 3 weeks. Before, during and after training twelve patients and twelve age-matched controls (the data from two controls had to be discarded later) took part in three functional magnetic resonance imaging (fMRI) sessions to assess training-related changes in the BOLD response in early visual cortex. Patients benefited from the training measurements as indexed by significant decrease (*p* = 0.001) in the stimulus onset asynchrony (SOA) between the presentation of the texture target on background and the visual mask, and in a significant location specific effect of the PRL with respect to hit rate (*p* = 0.014). The following trends were observed: (i) improvement in Vernier acuity for an eccentric line-bisection task; (ii) positive correlation between the development of BOLD signals in early visual cortex and initial fixation stability (*r* = 0.531); (iii) positive correlation between the increase in task performance and initial fixation stability (*r* = 0.730). The first two trends were non-significant, whereas the third trend was significant at *p* = 0.014, Bonferroni corrected. Consequently, our exploratory study suggests that training on the TDT can enhance eccentric vision in patients with central vision loss. This enhancement is accompanied by a modest alteration in the BOLD response in early visual cortex.

## INTRODUCTION

Visual performance in a variety of tasks, for example in the detection or discrimination of certain stimulus patterns, has been shown to improve with training. The results of this perceptual learning appear to have long lasting effects (e.g., Gibson, 1963; Goldstone, 1998; Fahle and Poggio, 2002; Seitz and Watanabe, 2005; Sagi, 2011; Frank et al., 2014). At the same time it often takes only hours or days of practice to enhance perceptual abilities dramatically. This has been shown for texture discrimination (Karni and Sagi, 1991), orientation discrimination (Schoups et al., 2001), spatial frequency discrimination (Fiorentini and Berardi, 1981; Sireteanu and Rettenbach, 1995), Vernier discrimination tasks (Poggio et al., 1992), and the discrimination of motion direction (Ball and Sekuler, 1982), among others.

Perceptual learning thus appears to provide an ideal approach to be used in clinical settings as well, in the attempt to improve the abilities of visually impaired persons. Recent studies have focused on amblyopia, where perceptual learning proved to improve vision in the amblyopic eye (e.g., Polat et al., 2004; Zhou et al., 2006; Levi and Li, 2009; Astle et al., 2010, 2011; Levi, 2012; Chung et al., 2012; Hussain et al., 2012). Other applications include applying perceptual learning in myopia and presbyopia (Polat, 2009; Polat et al., 2012), in adults with impairments in stereopsis (Ding and Levi, 2011) and in children with visual impairment (Huurneman et al., 2013) and developmental dyslexia (Gori and Facoetti, 2014). In patients with central vision loss, Chung (2011) used rapid serial visual presentation (RSVP) in an oral sentence-reading task to improve patients’ reading ability, a paradigm that has already been shown to improve reading speed in the peripheral visual field in both younger (Chung et al., 2004; Yu et al., 2010b) and older (Yu et al., 2010a) normally sighted adults. In Chung’s (2011) study, RSVP reading speed improved on average by 53%.

Central vision loss is often caused by atrophy of photoreceptor cells in the macula, as can be observed in age-related macular degeneration (AMD) or hereditary retinal dystrophies (juvenile form, JMD) like Stargardt’s disease or cone-rod dystrophy. Patients with central scotoma often develop eccentric viewing to cope with visual tasks like reading. The so-called “preferred retinal locus” (PRL) is a location in the eccentric visual field that is habitually used by MD patients as a pseudo-fovea (Bäckman, 1979; Timberlake et al., 1987; Whittaker et al., 1988; Guez et al., 1993; Fletcher and Schuchard, 1997). In this study, we trained AMD/JMD patients to perform a TDT (Karni and Sagi, 1991) with the target located at or near the PRL, with the aim to improve patients’ visual abilities at this specific location in their visual field. To investigate possible transfer effects to other tasks or abilities, we used the Freiburg Visual Acuity and Contrast Test (FrACT; Bach, 1996) before and after training. Possible effects on quality of life issues were assessed with the Visual Function Questionnaire VFQ-25 (Mangione et al., 2001).

We were also interested in the neural correlates of training using functional magnetic resonance imaging (fMRI). The neural correlates of perceptual learning are still not well understood. Results so far indicate an increase of the BOLD signal in primary visual cortex (Schwartz et al., 2002) with the training of a TDT. But it was also shown with fMRI that with repeated training learning is accompanied by an initial increase followed by a decrease in response (Yotsumoto et al., 2008). We observed a similar development in a recent study on the effect of trial-by-trial feedback on a challenging coherent-motion discrimination task (Goldhacker et al., 2014). In the initial phase of training we observed an increase in the fMRI-BOLD signal in primary visual cortex. With repeated training the BOLD signal in early visual cortex decreases. At the same time the performance of participants increases further or remains constant at a high level. We interpret this development in the BOLD signal over several measurements and days as an indication for neuroplastic changes in visual cortex as a consequence of intensive training. In the initial training phase, additional neural resources are recruited to learn the new perceptual task. After the task has been well practiced, neural processing becomes more automatic with equivalent high performance, thus less neural resources are needed. As suggested by Yotsumoto et al. (2008), the increase of brain activation in early visual cortex in the initial phase of learning could be mediated by an increase in the number or strength of synaptic connections, while the drop in activation at a later stage could be explained by synaptic downscaling after performance becomes saturated. This pattern is also in line with reports of participants, suggesting that they only guess at the beginning of training, while later they claim to “see” the differences in the stimuli clearly and almost without any effort (Goldhacker et al., 2014). Further studies show that perceptual learning can even lead to a parallelization of a visual conjunction search task which can only be solved in a serial manner initially (Frank et al., 2014).

In this study we explored the effects of perceptual learning in patients with central visual field loss. We investigate whether repeated intensive training can improve performance on the TDT, while altering the response of neurons in early visual cortex responsible for the processing of peripheral information. To test for the visual-field specificity of training, during fMRI we tested patients for targets located at their PRL or at a location opposite of the PRL (OppPRL). Comparison with an age-matched control group should indicate the extent to which this form of learning is specific for persons with central vision loss.

## MATERIALS AND METHODS

### PATIENTS AND CONTROL SUBJECTS

Eight patients with diagnosed AMD and five patients with juvenile macular dystrophy (JMD; i.e., three patients with cone-rod dystrophy and two patients with Stargardt’s disease) participated in the study (8 males, 5 females; mean age 63.8 years, range 47–79 years). Additionally twelve healthy age-matched control subjects took part in the experiment (4 males, 8 females; mean age 62.1 years, range 47–78 years). All participants signed an informed consent form prior to participating in the study and received modest monetary compensation for their participation. The study was approved by the Ethics Committee of the University of Regensburg and conducted in accordance with the ethical guidelines of the Declaration of Helsinki.

### CLINICAL CHARACTERISTICS AND VISUAL FIELD MEASUREMENTS

**Table 1** presents details on demographic characteristics of patients and controls, including the gender, age, diagnosis, duration of disease at time of study, study eye, scotoma size, visual acuity, position of PRL, and fixation stability in the study eye. The dominant eye was chosen as the study eye. Eye dominance was determined by a modified version of the A-B-C Vision Test (Miles, 1930; Porac and Coren, 1976), by aiming a distant target through an opening formed by their hands. The study eye of the controls was always the eye corresponding to the study eye of their age-matched patient. Since some of our measures were conducted in the Eye Hospital, fixation stability, and visual acuity could only be determined at the start of the study.

**Table 1:** Characteristics of patients (P1–P13) and controls (C1–C12) according to age, gender, diagnosis, duration of disease in years, study eye, decimal visual acuity, scotoma size (diameter in degrees visual angle), position of PRL (in degrees visual angle in x,y -coordinates with 0,0 put at central vision), and fixation stability (percentage of fixation in 2 and 4∘ visual angle around fixation target; patients fixated with their PRL, controls fixated with their fovea); m, male; f, female; Stargardt, Stargardt’s disease; OS, oculus sinister; OD, oculus dexter.

Patient Nr.	Age	Gender	Diagnosis	Duration of disease (in years)	Study eye	Decimal visual acuity (study eye)	Scotoma size in study eye (diameter in ∘ visual angle )	Position of PRL (in ∘ visual angle)		Fixation stability in study eye	
								x	y	2∘	4∘
P1	64	M	AMD	6	OS	0.1	10	-8	1	74	95
P2	64	M	AMD	7	OS	0.08	25	-4	-1.5	76	95
P3	79	M	AMD	9	OD	0.2	10	-6	3	89	100
P4	47	F	Stargardt	13	OD	0.05	15	-1.5	-6	95	100
P5	63	M	Cone-rod dystrophy	19	OD	0.1	15	0	-5	22	22
P6	57	M	Stargardt	18	OD	0.05	15	0	-6	33.7	66.7
P7	58	M	AMD	5	OD	0.02	20	-13	-0.5	10.6	28.3
P8	61	F	AMD	8	OS	0.3	10	-6	-3	60	100
P9	72	F	AMD	12	OS	0.1	10	5	0	88	98
P10	74	F	AMD	20	OS	0.2	10	4	-4	83	100
P11	63	F	AMD	8	OD	0.1	20	-6	3	90	99
P12	59	M	Cone-rod dystrophy	13	OS	0.1	10	-9	0	90	100
P13	69	M	Cone-rod dystrophy	59	OD	0.1	10	0	-4	100	100
C1	64	F	-	-	OS	0.9	-	-	-	100	100
C2	67	M	-	-	OS	1.0	-	-	-	100	100
C3	71	M	-	-	OD	1.0	-	-	-	100	100
C4	47	F	-	-	OD	1.0	-	-	-	100	100
C5	78	M	-	-	OD	0.9	-	-	-	100	100
C6	52	F	-	-	OD	1.6	-	-	-	100	100
C7	63	F	-	-	OS	0.8	-	-	-	100	100
C8	51	F	-	-	OS	1.4	-	-	-	100	100
C9	64	F	-	-	OS	1.2	-	-	-	99	100
C10	54	F	-	-	OD	1.0	-	-	-	99	100
C11	56	F	-	-	OS	1.4	-	-	-	100	100
C12	78	M	-	-	OD	0.9	-	-	-	85	97

**FIGURE 1:**
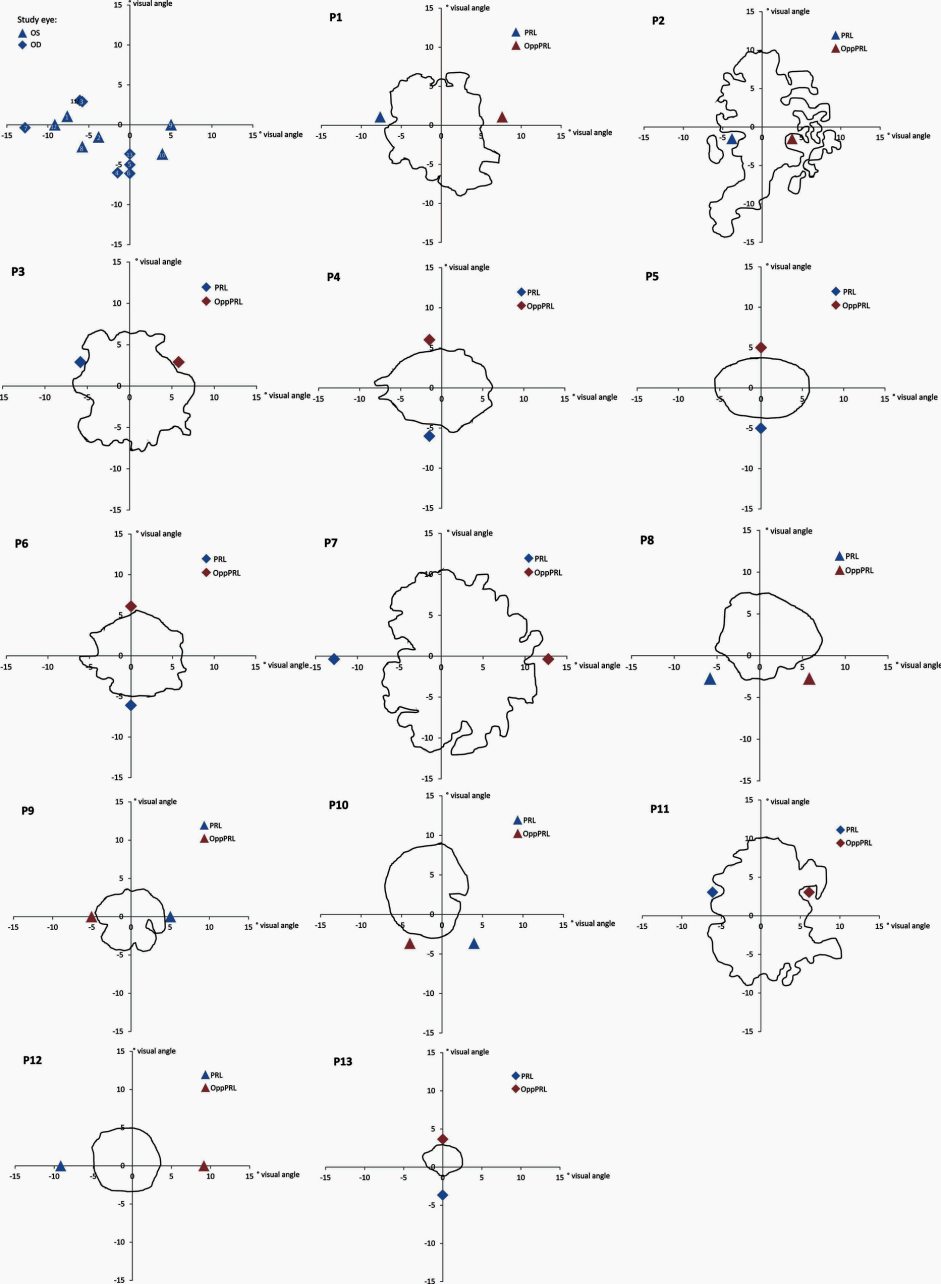
**Schematic depiction of positions of PRLs for all patients (upper left; blue triangles mark the left eye as study eye, blue diamonds mark the right eye as study eye, labeled with patient numbers 1–13) and schematic depictions of the shape of each patient’s scotoma as inferred from fundus photography (autofluorescence (P2, P3, P4, P5, P6, P8, P10, P11, P12, P13) or infrared reflection imaging (P1, P7, P9; blue symbols code the trained PRL position, red symbols code the untrained OppPRL position).** The x- and y-axis of the plots give the eccentricity in degrees of visual angle.

Best-corrected visual acuity was determined by using a Vision Screener (Rodenstock Rodavist 524/S1) and Eye Charts for distant visual acuity (Oculus Nr. 4616). Scotoma size was measured using kinetic Goldmann perimetry with the isopters III/4e, I/4e, I/3e, I/2e, and I/1e in all patients except patients P8, P10, and P11. Defined as edges of the scotomata, those points were marked, where isopter III/4e were no longer detected. Scotoma size is reported in **Table 1** as scotoma diameter in degrees of visual angle as an average and approximation of rounded vertical and horizontal dimensions. Reliability of the Goldmann perimetric measures depends on fixation stability. For patients P8, P10, and P11 no Goldmann perimetry was available. Scotoma size was inferred from fundus photography (autofluorescence imaging as described in Rosengarth et al., 2013) instead. Controls did not undergo Goldmann perimetry. **Figure 1** depicts the shape of each patient’s scotoma in the respective study eye as inferred from fundus photography. The techniques differ in principle as Goldmann perimetry provides direct visual field measures based on measures of visual function while fundus photography provides indirect evidence based on changes to fundus morphology.

As described in Rosengarth et al. (2013), we used a Nidek MP-1 microperimeter (Nidek Co, Japan) to measure fixation stability. Patients were requested to fixate (eccentrically) a red cross of 4° visual angle in diameter for approximately 30 s, whereas controls fixated the target with their fovea. The technique measures 25 samples per second, resulting in 750 fixation samples over 30 s. During the measurement the camera sometimes lost track of the subject’s eye. This can be due to eye blinks or fixation instability in the form of large saccades. The Nidek software records the time period that was measured and the proportion of the time span that was effectively tracked, as well as the percentages of fixation points that fell in a range of 2 or 4° diameter visual angle around the center of the fixation target, based on the time spans effectively tracked. Thus fixation stability can be overestimated by long or frequent time spans where the camera had lost track of eye position due to large saccades. To compensate for this we corrected the given fixation stability in the following way (see Plank et al., 2011): First we calculated the mean time span for which the camera lost track of eye position in the normally sighted control group, who fixated with their fovea. The resulting mean value of 9 s (SE = 3.0 s) yielded an estimate of the time that could be attributed to eye blinks. In a second step we subtracted this amount from the measured time, in which the camera had lost track of the eye of each patient. The individual difference between the measured time remaining and the effectively tracked time we attribute to large saccades. This time span was added to the effectively tracked time in each patient. On this basis we recalculated the percentages of fixation points falling in a range of 2 and 4° visual angle around the target for the patient group.

The Nidek MP-1 was also used to measure a microperimetry of 30° diameter around the patients’ PRL, for all patients except P8 and P11. Patients fixated a central cross with their PRL on intact retina and were instructed to press a button as soon as they perceived a target. We used “strategy-fast” with static light points of intensity 16 and 8 dB, maximal brightness of 127 cd/m^2^, that were presented for 200 ms each on a grid comprising the 30° of the visual field centered around the PRL.

The positions of PRLs were also assessed via the Nidek fundus images. They were later verified using a video eyetracker (High Speed Video Eyetracker Toolbox, Cambridge Research Systems, UK), while the patients fixated a target on a computer monitor. The distribution of positions of patients’ PRLs in the visual field is given in **Figure 1**.

### STIMULI AND TASK

**FIGURE 2:**
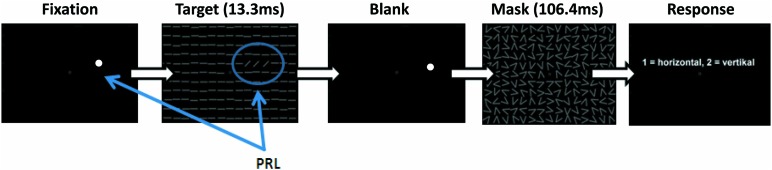
**Schematic depiction of a single trial in the training sessions.** While subjects were successfully fixating with their PRL (white dot) the target stimulus appeared for 13.3 ms followed by a mask, which was shown for 106.4 ms. The time between stimulus and mask (SOA) was adjusted to provide a constant hit rate of 70.7%. At the end of each trial subjects had to indicate by button press whether the three lines of the target formed a horizontal or vertical array.

Patients and controls were trained in a modified version of the TDT described by Karni and Sagi (1991). During training subjects were positioned with a distance of 60 cm in front of a 19-inch screen with a refresh rate of 75 Hz, while the luminance for black was 0.93 cd/m^2^ and for white 106 cd/m^2^. We used Matlab (version 7.12.0) and the Psychophysics Toolbox (Brainard, 1997) for programming the stimuli and the experimental design. Subjects were instructed to fixate with their individual PRL while controls had to hold their fixation in the center of the screen. To support patients’ fixation a white dot (0.75°) was placed at their individual PRL position. Controls fixated at a white circle (0.5° visual angle) at the center of the screen. During a trial, participants had to discriminate the global orientation (horizontal/vertical) of three tilted lines, located in their PRL, against a uniform background of horizontal lines (see **Figure 2**).

Stimulus size was increased in comparison to the original paradigm (Karni and Sagi, 1991), with a line length of 2° and line width of 0.3° visual angle. We did not scale the target elements nor the distractors in the background for different eccentric locations, since stimulus displays had to fit into a 30-degree diameter display. Target position was individually adjusted according to each patient’s PRL position. Each control subject was assigned to one particular patient and adopted that patient’s PRL position as target position in the task. On each trial, the target stimulus was shown for 13.3 ms, followed by a blank screen with variable stimulus onset asynchrony (SOA) and a mask stimulus (106.4 ms duration), after which the participants responded with a button press (two buttons on a standard keyboard; see **Figure 2**). In each block the SOA was adjusted by using an adaptive procedure (two–down, one-up), starting with a SOA of 492.1 ms, to determine the 70.7% correct threshold (Levitt, 1971). Initial step size was 53.2 ms, which was decreased by 13.3 ms (i.e., the duration of one frame on the display) after each turning point. A block stopped after 32 trials and the last measured SOA was taken as the 70.7% threshold of this block. In a pre-training session the initial individual SOA threshold was determined by running five experimental blocks. This initial SOA threshold was then used in all fMRI sessions. All patients and controls performed six training sessions on separate days over a period of approximately 3–4 weeks. Each session consisted of 20 blocks, each with 32 trials. One block took about 2 min, depending on individual SOA and reaction times, and each session lasted approximately 45 min.

### EYETRACKING DURING PSYCHOPHYSICAL MEASUREMENTS

A trial only could be evoked if fixation was stable, which was assured by an eye tracking system (resolution 0.05°, 250 Hz, High-Speed Video Eye-Tracker Toolbox, Cambridge Research Systems, Rochester, UK), thus the onset of trials could be delayed in case of unstable fixation. Calibration was done by controls with their fovea and by patients with their PRLs, resulting in a constant shift with respect to the position of the fovea. This constant shift, in coordinates of the individual PRL, was added as a correction factor to the tracked position of the eye.

### STIMULI AND TASK DURING fMRI

During the fMRI sessions, visual stimuli were projected onto a circular screen (31° visual angle in diameter at a distance of 60 cm) placed behind the head of the participant at the end of the scanner bore and visible via a mirror placed within the MRI head coil. Subjects underwent an fMRI session before training, after three training sessions and again after another three training sessions. The stimuli as described above appeared in a distance of 60 cm on the screen (luminance of the dark background was 1.7 cd/ m^2^, luminance of the white line elements was 193 cd/m^2^). The fMRI sessions differed somewhat from the training sessions, since the target stimulus was presented randomly in half of the trials in the PRL position and in half of the trials in the opposite hemifield (OppPRL), leading to slightly lower performance (see below). This was indicated by a brief color change of the white fixation dot before appearance of the target stimulus. In most subjects the fixation dot at the target location turned to red at the PRL or blue to indicate that the target would appear at the OppPRL. In some patients the color of the dot only changed when the target was to appear in the OppPRL because those patients had problems in differentiating the colors red and blue. This color cueing was kept constant for the matched control subjects. As in the training session patients fixated with their PRL, while control subjects kept fixation in the center of the screen. No eyetracker was used during fMRI, but fixation stability could be estimated from psychophysical test sessions. The SOA achieved before training sessions served as fixed SOA for all three fMRI sessions. At the beginning of a trial the dot changed its colour for 505.4 ms, followed by the target for 13.3 ms. After an individual SOA the mask was presented for 106.4 ms. Then a fixation pause with temporal jitter of 3–4 s succeeded before a new trial started. Each block consisted of 100 trials (50 PRL, 50 OppPRL), lasting for, on average, 8 min, again depending on individual SOA and reaction times. Three blocks were conducted in one fMRI session. The participants viewed all test stimuli in all training and testing situations monocularly with their study eye.

### FREIBURG VISUAL ACUITY AND CONTRAST TEST

Before and after training subjects’ visual acuities and contrast sensitivity at the trained position in the visual field were assessed by applying the FrACT^1^ (Bach, 1996) to monitor for possible improvements induced by training. Thereby the Landolt C contrast sensitivity test with 100 and 50 arcmin diameter, the contrast grating test and the Vernier test were chosen. Luminance linearization was applied as implemented in the software.

### VISUAL FUNCTION QUESTIONNAIRE

To assess the patients’ own perception of their visual function before and after perceptual learning we used the National Eye Institute’s VFQ-25 (Mangione et al., 2001) in its German translation.

### BEHAVIORAL DATA ANALYSIS

According to stimulus onset asynchronies obtained in the training sessions a 2 × 6 ANOVA for the factors group (patients, controls) and session (training session 1–6) was performed. To test explicitly for group differences in SOAs between training sessions 1 and 6, we applied two t-tests. For the fMRI sessions we conducted 2 × 2 × 3 ANOVAs related to the factors group (patients, controls), location (PRL, Opposite PRL) and session (before, during and after training) with respect to the dependent variables hit rate and reaction time. Additionally, we performed two 2 × 3 ANOVAs with the factors location (PRL, OppPRL) and session (before, during and after training), separately for each group, with respect to the dependent variables hit rate and reaction time. To test explicitly for group differences in hit rates between fMRI session 1 (before training) and 3 (after training), at the PRL and OppPRL, we applied four *t*-tests.

Additionally we performed correlation analysis between initial fixation stability, assessed before training started, and the development of hit rate and BOLD percent signal change in the PRL and OppPRL associated area in the early visual cortex.

For all ANOVAs, we corrected for violation of sphericity assumption if necessary by using Greenhouse–Geisser correction (*p* < 0.05). All statistical tests were performed using PASW 21 for Windows.

One patient (P13) was not able to participate in the fMRI sessions for physical reasons. We only included his behavioral values for the group analysis of the SOA measurement (see below). In total, data from 13 patients and 12 control subjects entered the SOA analysis of the behavioral data acquired during the training sessions. During the fMRI sessions, hit rate and reaction time were recorded in 11 patients and 10 control subjects. Behavioral data from one patient (P12) and two control subjects (C4 and C12) were lost due to technical problems with the response box.

According to the subtest of the FrACT and the VFQ a possible impact of training was assessed by paired *t*-tests (before and after training). Data from the FrACT were acquired in 13 patients and 12 control subjects. Data from one patient (P7) was excluded from the analysis of Landolt C contrast sensitivity, because he was not able to do the test. The data from another patient (P2) was excluded from the analysis of the Vernier test, owing to his inability to execute the Vernier test before training. Data from all 13 patients were available for the VFQ analysis.

### STRUCTURAL AND FUNCTIONAL MRI MEASUREMENTS

Magnetic resonance imaging scanning was performed with a 3-Tesla Allegra head scanner (Siemens, Erlangen, Germany) and a one-channel head coil. Functional whole-brain images were acquired interleaved with a T2^∗^-weighted gradient echo planar imaging (EPI) sequence (time-to-repeat, TR = 2 s; time-to-echo, TE = 30 ms; flip angle, FA = 90°) consisting of 34 transverse slices (voxel-size = 3 mm × 3 mm × 3 mm; field of view, FOV = 192 mm × 192 mm). In addition, we collected a high-resolution structural scan (160 sagittal slices each) with a T1-weighted, magnetization prepared rapid gradient echo (MP-RAGE) sequence (TR = 2.25 s, TE = 2.6 ms, FA = 9°, voxel size = 1 mm × 1 mm × 1 mm, FOV = 240 mm × 256 mm). The sequence was optimized for the differentiation of gray and white matter by using parameters from the Alzheimer’s disease Neuroimaging Initiative project^2^.

### MRI DATA ANALYSIS

Magnetic resonance imaging data analysis was performed with Statistical Parametric Mapping 8 (Wellcome Center of Neuroimaging, London^3^). First a temporal interpolation of the functional data using the slice time function in SPM8 was conducted. Afterward a motion correction over all sessions was applied to the functional images followed by co-registering each participant’s structural brain scan of the first session (before training) to the functional images. Then images were normalized to the MNI space, re-sampled to a 2 mm × 2 mm × 2 mm resolution and smoothed with a three-dimensional Gaussian kernel (full-width at half-maximum = 8 mm).

In the first-level statistical design the possible positions of the PRL and the OppPRL as prediction variable for each session were modeled separately and then convolved with the hemodynamic response function.

For a region-of-interest (ROI) analysis the SPM toolbox Marsbar was applied (Brett et al., 2002). A functional localizer was used to assess the individual representation area of the PRL, the OppPRL, and the fovea in the early visual cortex of the patients. Accordingly, during a separate fMRI scan contrast reverting checkerboard disks (size: 9° × 9° visual angle, presented with a reversal rate of 8 Hz) and chromatic images of everyday objects (e.g., animals, tools, vehicles, musical instruments; 7.3° × 7.3° visual angle) were visually presented on the individually determined position of the PRL, a location of the same eccentricity OppPRL and the fovea (corresponding to the scotoma region in the patients). For the control subjects the PRL/OppPRL coordinates of their age-matched patient were used. The PRL localizer scans were also conducted monocularly with the same study eye. The photographs used in the PRL localizer paradigm were collected from free Internet databases or taken by the authors. Stimuli were presented blockwise on a gray background, together with a baseline condition (gray background of medium luminance). The blocks were presented in four repetitions. Contrast reverting checkerboards and meaningful pictures were presented in the center, the PRL or the opposite PRL in separate blocks of 13 s each, the baseline condition (blank screen) in blocks of 18 s. In a block with meaningful pictures, the picture changed every 2.2 s without a gap, so that six different pictures were presented sequentially in each object block (for a detailed description see Rosengarth et al., 2013).

In a GLM analysis we modeled six regressors for the two types of stimuli (checkerboards, everyday objects) and the three locations (fovea, PRL, OppPRL) while the baseline condition (blank screen) served as an implicit baseline for the analysis to avoid an overspecification of the statistical design. Individually weighted T-maps for contrasts PRL > OppPRL and OppPRL > PRL were calculated. A sphere of 5-mm radius was placed on the voxel with the highest *t*-value of the resulting cluster in striate and extrastriate visual cortex. ROIs were always located in the hemisphere contralateral to the PRL/OppPRL location in the visual field. Since no explicit retinotopic mapping of visual area borders was conducted, we cannot separate these activations into the respective visual areas. These spheres served as ROIs for calculation of the individual percent signal changes in projection zones for the PRL and OppPRL in the visual cortex by applying these ROIs for the individual GLMs applied to the data of the main experiment.

The individual percent signal changes were integrated in a 2 × 2 × 3 factorial ANOVA with the factors group (patients, controls), location (PRL, OppPRL) and sessions (before, during, after training).

We also tested for the existence of a linear or quadratic trend in the factor session, with one-factorial ANOVAs, separately for each location (PRL, OppPRL) and group (patients, controls).

Because of technical issues two control subjects (C4 and C12) had to be excluded from the analysis of the fMRI data resulting in 12 patients and 10 controls for that analysis.

We also correlated patients’ fixation stability with the development of percent signal change of the BOLD response with the training.

## RESULTS

### BEHAVIORAL DATA

**FIGURE 3:**
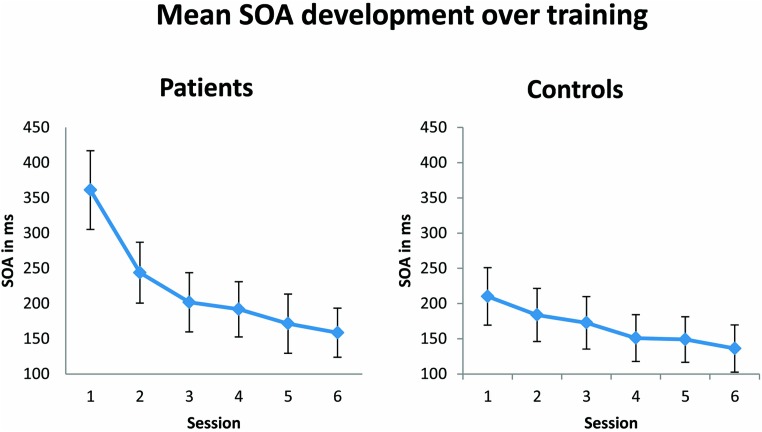
**Mean of stimulus onset asynchrony (SOA in ms) over six training sessions.** The SOA decreases significantly (*p* = 0.001) from the first to the last training session in both patient (*n* = 13) and control (*n* = 12) groups. Error bars show ±1 SE of the mean.

In agreement with the original results of Karni and Sagi (1991), during training patients and controls showed a training-induced improvement in performance, as reflected in a significantly decreasing SOA over the six training sessions [*F*(1,23) = 14.47; *p* = 0.001; see **Figure 3**). No significant effect of group was observed, suggesting that both patients and controls learned the task equally well. Although not significant, there was a trend toward an interaction between the factors group and session [*F*(1,23) = 3.5; *p* = 0.074]. This trend in the results appears to be due to the fact that patients started generally with higher SOAs, which were followed by a steeper decrease of SOAs over training compared to control subjects. Differences in SOAs in training session 1 between the patient and control group just failed to reach significance [*t*(23) = 2.2; *p* = 0.08, Bonferroni corrected for multiple comparisons]. SOAs in training session 6 were indistinguishable between patient and control group [*t*(23) = 0.46; *p* = 1.00].

**FIGURE 4:**
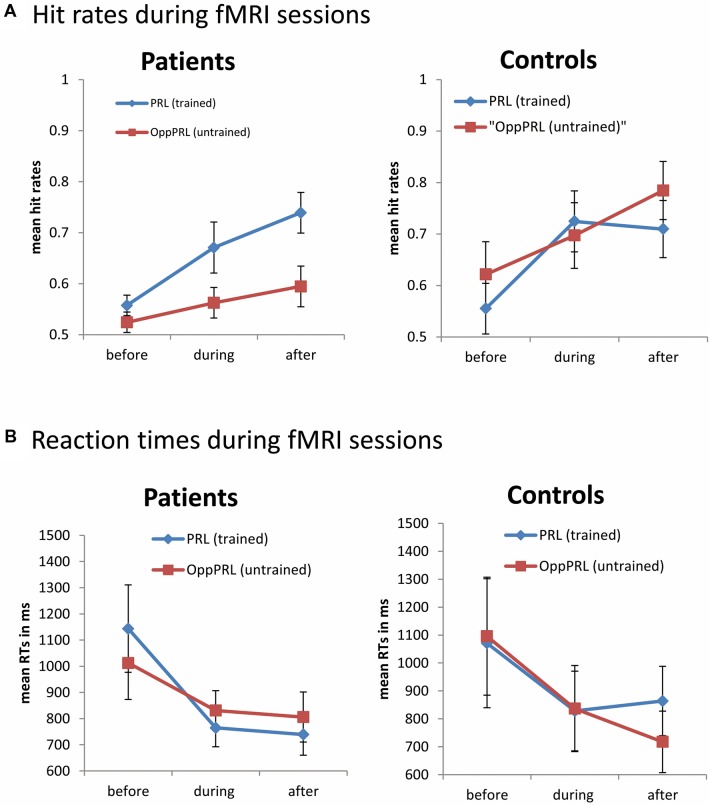
**Mean hit rates **(A)** and reaction times **(B)** in the PRL (trained location, blue symbols) and the OppPRL (untrained location, red symbols) for the patient (*n* = 11, left panel) and the control groups (*n* = 10, right panel) before the first, fourth and after the sixth training session.** Error bars show ±1 SE of the mean. Data were collected during the fMRI sessions, with individually fixed SOA. An omnibus ANOVA revealed a significant effect of session [*F*(1,19) = 13.6; *p* = 0.002] for the dependent variable hit rate, but no main effect of target location nor group. Additionally hit rates exhibited a significant interaction between target location (PRL, OppPRL) and group [*F*(1,19) = 4.6; *p* = 0.045]. For reaction times an omnibus ANOVA revealed again a main effect of session [*F*(2,38) = 6.6; *p* = 0.003], but no main effect for location nor group. Also no significant interactions were apparent.

During the fMRI sessions there was a significant effect of session [*F*(1,19) = 13.6; *p* = 0.002] for the dependent variable hit rate, but there was no effect of location nor group in the omnibus ANOVA (**Figure 4**, upper panel). Additionally hit rates exhibited a significant interaction between target location (PRL, OppPRL) and group [*F*(1,19) = 4.6; *p* = 0.045]. As can be seen in **Figure 4**, and was also tested by additional ANOVAs separately for patients and controls, there was a significant effect of target location (PRL, OppPRL) in the patient group [*F*(1,10) = 8.78; *p* = 0.014]. Accordingly, the hit rate was significantly higher when the TDT target was located in or near the PRL compared to when it was located in the opposite visual hemifield. The control group showed no significant location effect. Both groups, patients [*F*(2,20) = 9.5; *p* = 0.001] and controls [*F*(1,9) = 5.7; *p* = 0.04], showed a significant session effect, but no significant interactions.

For reaction times during the fMRI sessions we observed again a main effect of session [*F*(2,38) = 6.6; *p* = 0.003], indicating a decrease of reaction times with training, but no effect for location nor group (see **Figure 4**, lower panel). No significant interactions were apparent.

### TRANSFER OF TDT TRAINING

**FIGURE 5:**
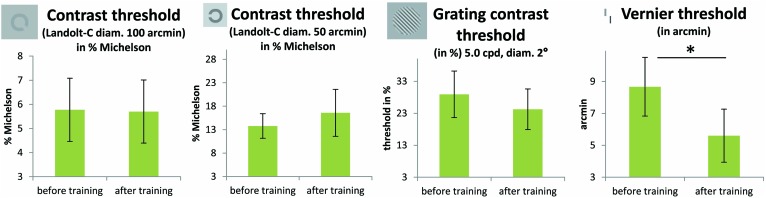
**Results from the FrACT (Bach, 1996).** Two Landolt-C tests (with 50 and 100 arcmin optotypes, in % Michelson contrast), a grating contrast test and a Vernier test were conducted. Improvements in patients’ performance with training were only apparent in the Vernier task [*t*(11) = 2.22; *p* = 0.048, not corrected for multiple comparisons; *p* = 0.2 Bonferroni corrected]. One patient (P2) was not able to perform the task before the perceptual training, but achieved a threshold of 5.58 arcmin in the task after the perceptual training. One patient (P7) was not able to perform the Landolt-C contrast sensitivity test, neither before nor after TDT training. Error bars show ±1 SE of the mean.

The results of the FrACT, analyzed with paired *t*-tests, showed a trend toward improvement of the Vernier task [*t*(11) = 2.22; *p* = 0.048, not corrected for multiple comparisons; otherwise *p* = 0.2, Bonferroni corrected] in the patient group (see **Figure 5**). Here it has to be noted that an additional patient (P2) was not able to perform the task before the perceptual training, but achieved a threshold of 5.58 arcmin in the task after the perceptual training. Contrast sensitivity measures did not differ before and after training, neither for Landolt-C with 100 arcmin diameter [*t*(11) = 0.05; *p* = 0.96] nor with 50 arcmin diameter [*t*(11) = -0.5; *p* = 0.62], nor for the contrast grating test [*t*(12) = 0.85; *p* = 0.41, all *p*-values not corrected for multiple comparisons]. One patient (P7) was not able to perform the Landolt-C contrast sensitivity test, neither before nor after TDT training. The control group did not improve significantly with training in any subtests of the FrACT.

Compared to values acquired before TDT training, patients yielded higher scores in the VFQ in the category of social functioning [*t*(12) = 2.79; *p* = 0.016, not corrected for multiple comparisons; otherwise *p* = 0.18, Bonferroni corrected] after training. All other scales showed no significant differences before and after training.

### EFFECT OF FIXATION STABILITY IN BEHAVIORAL DATA

**FIGURE 6:**
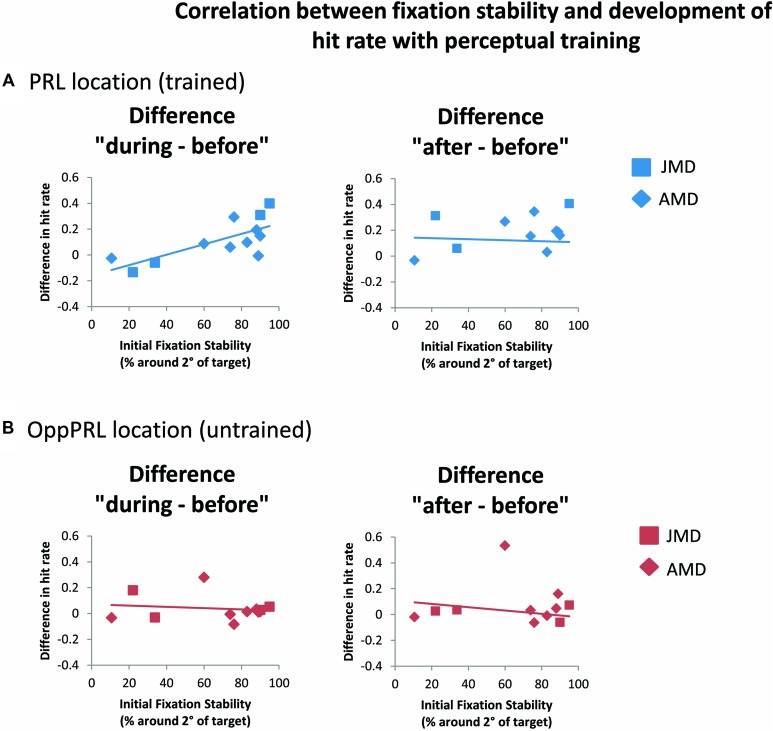
**Correlations between the difference in hit rate (before the first and fourth training session (“during”), left panel, as well as before the first and after the sixth training session (“after”), right panel, and initial fixation stability for the trained PRL location **(A)** or the untrained OppPRL location **(B)** for patient data only.** Squares correspond to values from JMD patients, diamonds to those from AMD patients. Correlation coefficients for the trained PRL location are *r* = 0.730 (*p* = 0.007; *p* = 0.014, Bonferroni corrected) for the difference in hit rate “during–before,” and *r* = 0.361 (*p* = 0.275) for the difference in hit rate “after–before.” For the untrained OppPRL location correlation coefficients are *r* = -0.145 (*p* = 0.652) for the difference in hit rate “during–before,” and *r* = 0.015 (*p* = 0.966) for the difference in hit rate “after–before.”

When we correlated fixation stability before training (percentage of fixations around 2° visual angle of the fixation point) and the development of hit rate in the patient group, separately for the trained PRL and the untrained OppPRL, we found a significant positive correlation with difference in hit rate between before and during training, but only for the trained PRL (*p* = 0.007, not corrected for multiple comparisons; otherwise *p* = 0.014, Bonferroni corrected; see **Table 2**; **Figure 6**). A correlation between fixation stability and development of reaction times in the patient group, separately for PRL and OppPRL, revealed no significant results.

**Table 2:** Correlation coefficients (*r*) and *p*-values (*p*; not corrected for multiple comparisons) between initial fixation stability (percentage of fixations within 2∘ of fixation target) and difference in mean percent signal change (upper rows) before the first and fourth training session, as well as before the first and after the sixth training session for PRL and OppPRL target locations.

Delta % signal change	Difference “during–before”		Difference “after–before”	
	*r*	*p*	*r*	*p*
PRL	0.155	0.629	0.531	0.075
OppPRL	0.042	0.896	0.444	0.148
Delta Hit rate				
PRL	**0.730**	**0.007**	0.361	0.275
OppPRL	-0.145	0.652	0.015	0.966

*Significant values are shown in bold font. These values are based on patient data only (*n* = 12).*

### fMRI DATA

**FIGURE 7:**
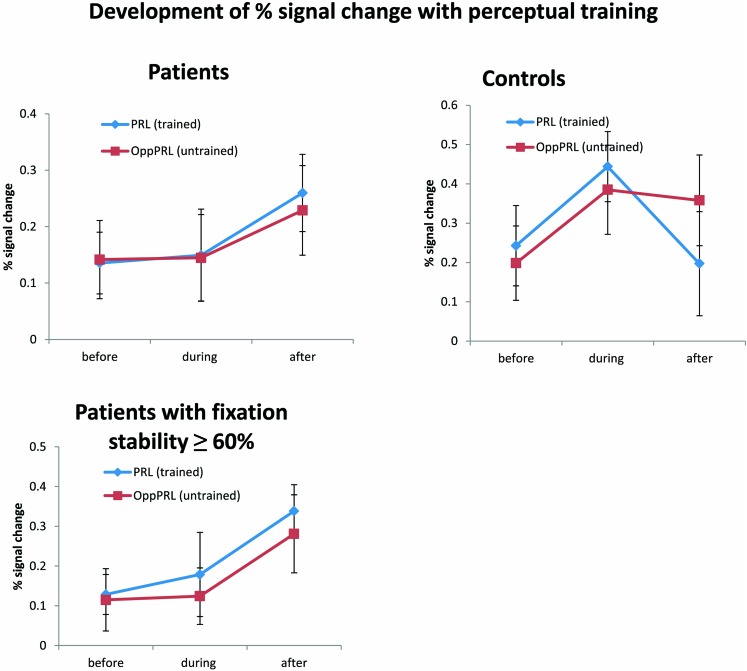
**Mean of percent signal change in the projection zones in the early visual cortex of the PRL (trained location, blue symbols) and the OppPRL (untrained location, red symbols) for patients (*n* = 12) and controls (*n* = 10; upper row) before the first, fourth and after the sixth training session.** An omnibus ANOVA revealed no significant effects. The lower row shows these values for those patients who exhibited fixation stability over 60% (*n* = 9). Here, a repeated-measures ANOVA within the patient group revealed a significant effect of training session (*p* = 0.038). Error bars show ±1 SE of the mean.

Patients exhibited a trend for increased percent signal changes from the second to the third fMRI session which was similar for the PRL and OppPRL projection zones in the early visual cortex, but which failed to reach statistical significance (see **Figure 7**, upper panel). While patients showed no obvious change in percent signal change from the first to the second fMRI session control subjects revealed an increase of percent signal change from the first to the second fMRI session in both the trained and untrained projection zones in the early visual cortex. From the second to the third fMRI session, patients exhibited a modest increase in BOLD signal, whereas controls showed a decrease for the signal in the trained PRL associated area and a stabilization of the OppPRL associated area.

A repeated-measures ANOVA revealed no significant effect of session [*F*(2,40) = 1.7; *p* = 0.20], nor an effect of location [*F*(1,20) = 0.02; *p* = 0.89] or group [*F*(1,20) = 2.09; *p* = 0.16] in the omnibus ANOVA. Also no interactions were significant. One-factorial ANOVAs for the factor session for the patients and controls separately with target locations either PRL or OppPRL indicated a marginally significant quadratic trend (blue line in **Figure 7**, upper right panel) for the control group [*F*(1,9) = 5.05; *p* = 0.05, not corrected for multiple comparisons; otherwise *p* = 0.1, Bonferroni corrected]. Moreover, a non-significant linear trend (blue line in **Figure 7**, upper left panel) was apparent for the patient group [*F*(1,11) = 3.04; *p* = 0.11, not corrected for multiple comparisons; otherwise *p* = 0.22, Bonferroni corrected] with respect to the effect of training (sessions performed before, during and after) on percent signal change in the PRL projection zone in the early visual cortex. For the OppPRL condition (red lines in **Figure 7**, upper left and right panel), no such trends were observed (*p* = 0.42 and *p* = 0.35, respectively, not corrected for multiple comparisons).

### EFFECT OF FIXATION STABILITY IN fMRI DATA

**FIGURE 8:**
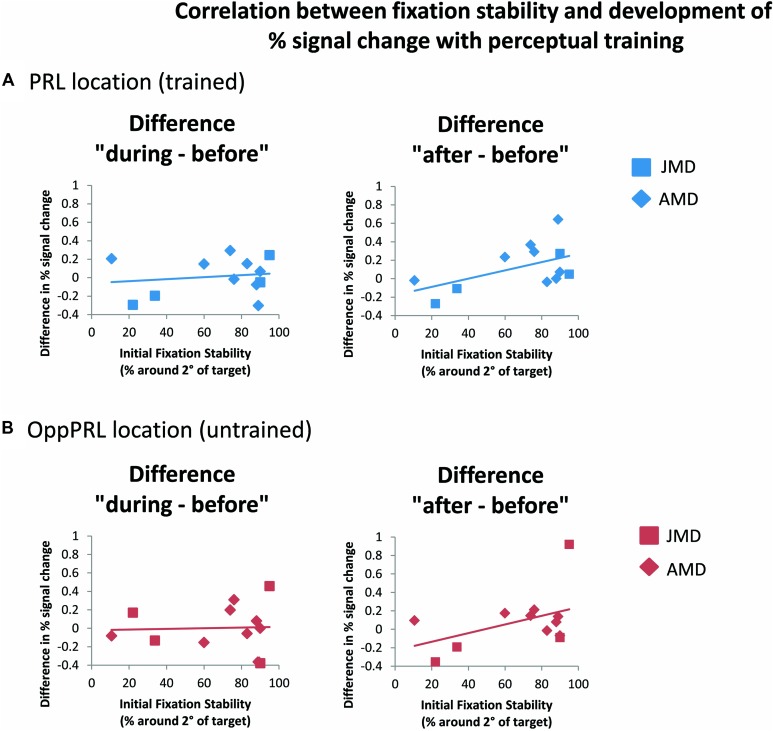
**Correlations between the difference in percent signal change (before the first and fourth training session (“during”), left panel, as well as before the first and after the sixth training session (“after”), right panel, and initial fixation stability for the trained PRL location **(A)** or the untrained OppPRL location **(B)** for patient data only (*n* = 12).** As in **Figure 6**, squares correspond to values from JMD patients, diamonds to those from AMD patients. Correlation coefficients for the trained PRL location are *r* = 0.155 (*p* = 0.629) for the difference in percent signal change “during–before,” and *r* = 0.531 (*p* = 0.075) for the difference in percent signal change “after–before.” For the untrained OppPRL location correlation coefficients are *r* = 0.042 (*p* = 0.896) for the difference in percent signal change “during–before,” and *r* = 0.444 (*p* = 0.148) for the difference in percent signal change “after–before.”

When we correlated fixation stability (percentage of fixations around 2° visual angle of the fixation point) and the development of BOLD signal in visual cortex, we found a positive correlation between fixation stability and difference in percent signal change before and after training that just failed to reach significance (*p* = 0.075, not corrected for multiple comparisons; otherwise *p* = 0.15, Bonferroni corrected; see **Table 2**; **Figure 8**).

As becomes evident from **Figures 6** and **8**, a gap in fixation stability could be observed between three patients (P5, P6, and P7) with fixation stability <40% and the remaining patients, who exhibit more stable fixation (≥60%). After excluding the data from these three patients with fixation stability <40%, an ANOVA of BOLD percent signal change revealed a significant effect of training session [*F*(2,16) = 4.1; *p* = 0.038; see **Figure 7**, lower panel] within the patient group.

## DISCUSSION

In this study we investigated whether patients with central vision loss can benefit from perceptual learning. We wanted to determine whether patients with central vision loss can be efficiently trained at their eccentric PRL to perform a challenging TDT and if such a learning effect might be reflected in fMRI-BOLD signal changes in the respective projection zone in early visual cortex. Further we investigated whether the gains accruing via TDT training at the PRL could generalize to other aspects of visual performance and vision-related aspects of quality of life.

Both patients and control subjects exhibited a typical learning effect on the TDT which was indicated by a significant reduction in SOA in both groups. This result is consistent with the classical findings of Karni and Sagi (1991), Schwartz et al. (2002), or Yotsumoto et al. (2008). Behavioral data acquired during fMRI indicated a significant effect of training on hit rates and reaction times. Considering the two groups (patients, controls) separately there was a significant effect of training in the patient group for the factor location (PRL, OppPRL), which was not the case for the control group. We further observed a significant interaction between target location and group with respect to hit rates (see **Figure 4A**). Before training patients showed similar hit rates for targets presented at the PRL and OppPRL locations in the visual field. During training their hit rate increased for targets presented at the PRL compared to when they were presented at the location OppPRL. In contrast control subjects showed also an increase in hit rates with training but no difference between the trained and untrained locations. One explanation for this finding could be that patients use their PRL additionally in their daily life which could influence the training procedure and efficiency. Therefore it might also be more intuitive for the patients to train on targets presented in their PRL since the PRL functions as a pseudo fovea, which is not the case for the control subjects.

In the fMRI results, we found neither a significant effect of session, nor of location nor of group in the omnibus ANOVA. We could observe a linear trend for the factor “training session” at the signal in the PRL projection zone in early visual cortex in the patient group while the control group seemed to exhibit a quadratic trend in that area. McGovern et al. (2012) claim that the low signal change which is sometimes found in studies dealing with perceptual learning in early visual areas (e.g., Ghose et al., 2002) might not be associated with the increase of performance directly. This suggestion also seems to hold here, since we could find clear learning effects according to SOA, hit rates and reaction times but only subtle changes of the amplitude of the BOLD signal with training. McGovern et al. (2012) argue that probably more brain areas than the early visual cortex might be involved in perceptual learning. The linear trend in patients of the signal in the PRL associated area in early visual cortex according to training is expressed in an increase of signal from the second to the third fMRI session. When we restricted our analysis to the subgroup of patients with high fixation stability (≥60%), we found a significant increase of BOLD response in early visual cortex with training. This result is consistent with several other studies which report an increase in neural signal in early visual cortex with training. Frank et al. (2014) show also an increase of percent signal change over learning sessions while subjects trained in a challenging perceptual learning task. The time course of the neural signal referring to the trained location in early visual cortex in the control group follows the pattern observed in the study by Yotsumoto et al. (2008) who also used a TDT. Similar to the trend of the present results for the control group, they found an increase of signal from the pre-training session to the second fMRI session followed by a slight decrease of signal in the post-training session. Interestingly this was only the case for the PRL associated area in early visual cortex where subjects received training and not for the untrained OppPRL associated area. As described in the Introduction, the increase in BOLD signal observed in the initial phase of learning suggests the recruitment of respective brain areas in early visual cortex (Yotsumoto et al., 2008). The decline in the BOLD signal would accordingly correspond to a consolidation process. In our study the control subjects appeared to have reached the consolidation phase already after the first post-training session, while patients still showed an increase in BOLD-signal up to the second post-training session.

Considering the patients’ fixation stability there was on the one hand a significant positive correlation between fixation stability and hit rate (difference during and before training) if the target appeared at the position of the PRL and on the other hand a positive correlation between fixation stability and percent signal change (difference after and before training) if the target was located in the PRL projection zone in early visual cortex, that just failed to reach significance. There was further a significant effect of session when three patients, who exhibited extremely poor fixation stability, were omitted from analysis. This finding suggests that fixation stability might be a prerequisite for a successful learning curve in perceptual learning. Moreover, other visual tasks seem to be affected by fixation stability. Plank et al. (2013) reported that patients suffering from hereditary macular dystrophies (JMD) with stable eccentric fixation performed better in a visual search task than patients with less stable eccentric fixation. Interestingly this was also the case, if the target stimuli were not in or near the position of the PRL. Fixation stability has also usually been shown to be positively correlated with reading speed in patients with central vision loss (e.g., Sunness et al., 1996; Trauzettel-Klosinski and Tornow, 1996; Nilsson et al., 1998; Nilsson et al., 2003; Crossland et al., 2004; Rubin and Feely, 2009). Please note that, since eye movements were not recorded during fMRI sessions, we had to assume that the level of fixation stability measured during psychophysical testing was also evident during fMRI testing.

The FrACT sensitivity (Bach, 1996) revealed a training associated improvement in the patient group for the Vernier subtest. However, it should be noted that the significance level of this effect does not survive correction for multiple testing, suggesting that caution must be exercised here and that further studies are warranted. The other tasks seemed not to be influenced by the training intervention. The reason for the marginal improvement in the Vernier task might be due to the similarity among the stimuli in the TDT and the Vernier task.

With respect to the transfer of TDT training the findings reported above suggest that caution should be exercised when interpreting their implications with respect to potential application in visual rehabilitation. Obviously studies with larger patient samples are required that assess the amount of transfer of perceptual training at the PRL to other visual functions. The addition of a “sham” training group would establish the extent to which placebo effects influence perceptual learning in select patient groups. With respect to the effects of oculomotor and eccentric-fixation training in a similar patient group, we could recently rule out that the beneficial effects of training could be explained by a general placebo effect (Rosengarth et al., 2013).

Earlier studies have pointed to a persistence of perceptual learning effects. Polat et al. (2004) found a two to fourfold increase in contrast sensitivity in the amblyopic eye of trainees 12 months after training on a flanker-task had ended. Our group has recently shown that in healthy participants the effects of perceptual learning of a difficult conjunction visual search task are still evident at 9-month follow-up (Frank et al., 2014). We are currently retesting the patients and controls of the present study with respect to this aspect of the results (Plank et al., unpublished observations).

With respect to the results of the VFQ, patients exhibited higher scores after training on the category of social functioning, which considers personal contact und communication with other people. Šiaudvytytė et al. (2012) report differences in quality of life of AMD patients compared to age-matched control subjects in several categories of the VFQ including social functioning. Possible implications of these trends require further investigation in larger patient samples.

## CONCLUSION

In this study we trained patients with central vision loss in a TDT, with the target appearing on their respective PRL, and compared their results to an age-matched normal sighted control group. We were also interested in the neural correlates of the learning process in the visual cortex. Although the task appeared to be more difficult for the patient group than for the control group, patients were able to do the task and showed significant learning effects. Patients with stable eccentric fixation showed better performance accompanied by a larger increase in BOLD-signal in the PRL projection zone of the early visual cortex. Owing to our strict inclusion and exclusion criteria with respect to disease manifestation in the study and companion eye of our patients, our results are limited to the present patient sample, thereby demanding further verification of beneficial effects of perceptual training in patients with different forms of macular disease. Nevertheless, the present results support the idea that perceptual learning can improve the efficient use of the PRL location in patients with central vision loss.

## Conflict of Interest Statement

The authors declare that the research was conducted in the absence of any commercial or financial relationships that could be construed as a potential conflict of interest.

## Footnotes

1 http://www.michaelbach.de/fract/download.html

2 http://adni.loni.ucla.edu/

3 http://www.fil.ion.ucl.ac.uk/spm/
